# Adaptation of an evidence-based intervention to promote colorectal cancer screening: a quasi-experimental study

**DOI:** 10.1186/1748-5908-9-85

**Published:** 2014-07-02

**Authors:** Shin-Ping Tu, Alan Chun, Yutaka Yasui, Alan Kuniyuki, Mei-Po Yip, Vicky Taylor, Roshan Bastani

**Affiliations:** 1 Department of Medicine, Virginia Commonwealth University, 1201 East Marshall Street, Richmond, VA 23298, USA; 2 Department of Health Services, University of Washington, Seattle, WA, USA; 3 International Community Health Services, 720 8th Avenue South, Seattle, WA 98104, USA; 4 School of Public Health, University of Alberta, 3-27 University Terrace, 8303-112 St, Edmonton T6G 2T4, Alberta, Canada; 5 Department of Epidemiology, University of Washington, 4333 Brooklyn Avenue NE, Seattle WA98195, USA; 6 Department of Medicine, Harborview Medical Center, University of Washington, 325 Ninth Avenue, Seattle, WA 98104, USA; 7 Division of Public Health Sciences, Fred Hutchinson Cancer Research Center, 1100 Fairview Ave. N., M3-B232, P.O. Box 19024, Seattle, WA 98109-1024, USA; 8 Department of Health Services, UCLA School of Public Health, 650 Charles Young Dr., A2-125 CHS, Box 956900, Los Angeles, CA 90095, USA

**Keywords:** Adaptation, Implementation, Evidence-based intervention

## Abstract

**Background:**

To accelerate the translation of research findings into practice for underserved populations, we investigated the adaptation of an evidence-based intervention (EBI), designed to increase colorectal cancer (CRC) screening in one limited English-proficient (LEP) population (Chinese), for another LEP group (Vietnamese) with overlapping cultural and health beliefs.

**Methods:**

Guided by Diffusion of Innovations Theory, we adapted the EBI to achieve greater reach. Core elements of the adapted intervention included: small media (a DVD and pamphlet) translated into Vietnamese from Chinese; medical assistants distributing the small media instead of a health educator; and presentations on CRC screening to the medical assistants. A quasi-experimental study examined CRC screening adherence among eligible Vietnamese patients at the intervention and control clinics, before and after the 24-month intervention. The proportion of the adherence was assessed using generalized linear mixed models that account for clustering under primary care providers and also within-patient correlation between baseline and follow up.

**Results:**

Our study included two cross-sectional samples: 1,016 at baseline (604 in the intervention clinic and 412 in the control clinic) and 1,260 post-intervention (746 in the intervention and 514 in the control clinic), including appreciable overlaps between the two time points. Pre-post change in CRC screening over time, expressed as an odds ratio (OR) of CRC screening adherence by time, showed a marginally-significant greater increase in CRC screening adherence at the intervention clinic compared to the control clinic (the ratio of the two ORs = 1.42; 95% CI 0.95, 2.15). In the sample of patients who were non-adherent to CRC screening at baseline, compared to the control clinic, the intervention clinic had marginally-significant greater increase in FOBT (adjusted OR = 1.77; 95% CI 0.98, 3.18) and a statistically-significantly greater increase in CRC screening adherence (adjusted OR = 1.70; 95% CI 1.05, 2.75).

**Conclusions:**

Theoretically guided adaptations of EBIs may accelerate the translation of research into practice. Adaptation has the potential to mitigate health disparities for hard-to-reach populations in a timely manner.

## Background

Cancer causes significant morbidity and mortality in the United States [1]. In 2014, an estimated 50,310 people will die as a result of colorectal cancer (CRC), the second leading cause of cancer-related deaths [2]. Regular screening for CRC is an effective method to lower cancer-related morbidity and mortality [3]. When CRC is detected at an early stage, five-year survival rates exceed 90% for those with localized disease, compared to 12% for those with distant metastases [4].

The US Preventive Services Task Force (USPSTF) recommends CRC screening for average-risk individuals aged 50 to 75 years by using one of the following modalities: annual high-sensitivity fecal occult blood test (FOBT), sigmoidoscopy every five years combined with FOBT every three years, or colonoscopy every 10 years [3]. Despite the availability of these modalities, CRC screening remains underused. According to the 2010 National Health Interview Survey, CRC screening rates were 58.6% in the overall population, well below the Healthy People targeted goal of 70.5% for 2020 [5].

Populations that are disproportionately under-screened, including many racial and ethnic minorities, urgently need organized efforts to promote CRC screening [6]. Because of advances in screening and treatment, the incidence and mortality of CRC have been declining over the last 25 years [7,8]. Unfortunately, this decline has not been shared equally by all groups, resulting in a growing racial and ethnic survival gap over the same 25-year period [8-10]. CRC screening rates for Whites (59.8%) are consistently higher than those of minority populations: African Americans (55%); American Indians and Alaskan Natives (49.5%); Asian Americans (46.9%); and Hispanics (46.5%) [5]. Among immigrants, CRC screening rates have decreased since 2008 for persons living in the US less than 10 years from 25.7% to 21.3% [5,6]. Predictors of low screening adherence among immigrants include low economic status, language barriers, lack of knowledge, lack of a regular source of health care, and lack of insurance [11,12].

Numerous barriers delay the dissemination of evidence-based interventions (EBIs) into clinical practices and communities [13]. Experts have underscored the tension between fidelity to implementing EBIs and adapting EBIs for the ‘real world’ [14]. In fact, EBIs are rarely implemented in the exact manner of the original trial [14]. Adaptations are often needed to ensure that interventions are feasible and ‘fit’ the target population and setting [14,15].

To promote effective dissemination and implementation, Allen *et al.* have underscored the need for evidence-based strategies to guide the adaptation of interventions in order to maintain high fidelity and minimize loss of intervention impact [14]. Although most available adaptation literature focuses on cultural modification of EBIs [14,16], two publications from the past decade expand their scope beyond this domain [17,18]. Castro *et al.* identified two basic forms of adaptation: changes in content and changes in the characteristics of the delivery person [17]. Backer presented four approaches to adaptation: adding or deleting program components; changing program components or content; changing the process or intensity of implementation; and making cultural modifications [18].

To accelerate the transfer of EBIs into clinical practice, we studied the adaptation of an EBI as guided by Diffusion of Innovations Theory to expand its reach within the diverse patient population of a community health center [19,20]. In terms of the theory’s Innovation-Decision Process, Rogers emphasized the importance of the communication channel—the means by which a message travels from a source to a receiver. Although mass media channels that can reach a large audience are important in Rogers’ knowledge stage, interpersonal channels that involve face-to-face exchanges are more important to most people in the persuasion stage. Interpersonal channels are also more effective than mass media in forming and changing strongly held attitudes [21].

The original EBI had a strong intervention effect (adjusted OR = 5.91; 95% CI = 3.25, 10.75) and consisted of a culturally and linguistically appropriate clinic-based educational program to promote CRC screening among Chinese immigrants using small media, a bicultural and trilingual (English, Cantonese, and Mandarin) Chinese health educator, and provision of FOBT kits [22]. Based on Rogers’ five influential attributes of innovation (relative advantage, compatibility, complexity, triability, and observability), we adapted the intervention agent to achieve a broader reach within the target population. In this article, we report findings from a quasi-experimental study to evaluate the adapted EBI.

## Methods

This research was conducted in collaboration with International Community Health Services (ICHS), a community health center serving predominantly limited English-proficient (LEP) Asian immigrants residing in the metropolitan area of Seattle, Washington. ICHS provides comprehensive primary care services at two sites: Holly Park Clinic and International District Clinic. All study procedures were approved by the Institutional Review Board of the University of Washington in Seattle.

Usual care for CRC screening at ICHS consisted primarily of FOBT at the time of the proposed research, and must be ordered by primary care providers (physicians, physician assistants, and nurse practitioners). Medical assistants (MAs) implemented all orders for FOBT and instructed patients to return completed FOBT cards to the clinic laboratory for processing and recording results in the ICHS electronic medical records (EMR) system. With respect to endoscopic screening, ICHS has the capacity to perform sigmoidoscopy, but this modality became infrequently used, and ICHS providers refer patients to gastroenterologists for screening colonoscopy primarily at patients’ preference and for diagnostic evaluation.

Chinese immigrants represent the largest patient population at ICHS, followed by Vietnamese patients [23]. Given commonalities in the traditional health beliefs of the Chinese and Vietnamese, both in Asia and in the US, replication of our intervention materials for Vietnamese Americans was both feasible and appropriate [24-29].

We adapted our original EBI with: MAs serving as the intervention agents instead of a health educator; no FOBT kits provided by the MAs as consistent with ICHS procedures; and a series of brief in-service presentations, each about 10 to 15 minutes long, to the MAs. During the in-service presentations, MAs were asked to distribute the intervention materials (translated into Vietnamese) to Vietnamese patients who appeared to be age eligible and specifically informed them that they were not expected to provide health education. We conducted a total of 15 presentations to MAs and two to all the staff at the intervention clinic. The adapted intervention period lasted two years, from 1 March 2009 to 28 February 2011.

As summarized in Table 1, adapting our intervention agent from a health educator to MAs provided economic advantages as well as better compatibility, simplicity, and trialability. Using less specialized MAs represents an economic advantage over using health educators. As integral members of the clinical team [30], MAs are involved in many direct patient interactions—for example, when they check patients in, MAs ask patients about problems to be addressed by providers, obtain vital signs, and coordinate necessary tasks at the conclusion of the medical visit. For optimal clinic operations, the number of MAs in a clinic must be proportional to the number of patients seen. By virtue of their numbers and the kinds of interactions in which they engage, MAs are better placed to reach patients and disseminate information than are health educators. For all these reasons, MAs are more compatible with the staffing needs of a busy clinic.

**Table 1:** Adaptation of the evidence-based intervention as guided by diffusion of innovations theory

**Diffusion of innovation attributes**	**Health educator**	**Medical assistant**
**Relative advantage**		
Economic	No	Yes
Social	NA	NA
Information	Yes	Yes
Health promotion	Yes	Yes
**Compatibility**		
Organizational level		
Staffing	No	Yes
Individual level		
Culture	No	No
Language	No	No
**Simplicity**		
Delivery in clinic setting	No	Yes
Guidelines	No	Yes
**Trialability**	No	Yes
**Observability**	Yes	Yes

### Design

Using a quasi-experimental study design, we examined naturalistic cross-sectional data of CRC screening adherence rates among eligible Vietnamese patients at the intervention and control clinics, before and after our 24-month intervention. We extracted baseline data as of 1 March 2009 and follow-up data from the ICHS NextGen Enterprise Practice Management and EMR system as of 28 February 2011, then compared these two cross-sectional data to infer the intervention effect.

Baseline demographic, insurance, primary care provider, clinic visit, and CRC screening data were extracted and have been described in a prior paper [31]. In accordance with USPSTF guidelines for CRC screening, our study sample consisted of Vietnamese patients who were 50 to 75 years old 12 months before the date of our data extraction (*i.e.*, 51 to 76 years of age on date of data extraction) [3]. We restricted our analyses to active patients, defined as patients who had at least one clinic visit in the previous 24 months. Using ICD 9 codes, we excluded patients with a prior diagnosis of CRC or inflammatory bowel disease.

For our analyses, we initially reviewed patient visit dates to better understand the stability of the targeted Vietnamese population at each clinic over our two assessment periods. Subsequently, we examined the data for differences in key patient characteristics between clinic sites at each time point, to identify potential imbalances in the study sample that might alter our interpretation of the data. Chi-square tests of homogeneity (and Fisher’s exact test, as needed) were performed to examine associations between CRC screening (FOBT; flexible sigmoidoscopy plus FOBT; colonoscopy; overall adherence) and clinic site, demographics, insurance status, continuity of care, comorbidities, and provider characteristics. Per current USPSTF guidelines, we defined adherence to CRC screening as meeting one of the following criteria: three FOBT cards within the past year; flexible sigmoidoscopy within the past five years plus three FOBT cards in the past three years; or colonoscopy within the past 10 years [3]. Adherence percentage was calculated as the percentage of patients in the target population who were adherent to screening.

For the main analysis, we examined the effectiveness of the intervention among targeted Vietnamese patients by determining changes in CRC screening adherence rates over time between the two clinics. We used generalized linear mixed models (GLMMs) to model CRC screening adherence among all eligible Vietnamese patients at both clinics, pre- and post-intervention. GLMM analyses were performed with the use of a logit link with two random effects to account for clustering of patients under primary care providers (27 providers in total) and within-patient correlation for patients who appear in both cross-sectional time points. Our GLMM models incorporated the study design factors of time (baseline, follow-up), clinic (intervention clinic, control clinic), and their interaction (time × clinic). Additional adjustments were made for age, gender, insurance status, type of primary care provider, language concordance with primary care provider, and continuity index. Percentages, odds ratios with associated 95% confidence intervals, and p-values are presented to describe and test the differences over time in CRC screening adherence rates between the two clinics.

In a secondary analysis, we stratified our study sample into two groups: patients who were non-adherent to CRC screening at baseline and those who were adherent at baseline. We analyzed these two groups separately for adherence at post-intervention using GLMMs. All statistical analyses for this study were conducted using SAS Version 9.2, SAS Institute Inc., Cary, NC.

## Results

At baseline, 1,016 Vietnamese patients and at post-intervention 1,260 met the inclusion criteria for age and for having at least one medical visit in the previous 24 months. Among patients at baseline, 753 remained post-intervention (449 patients in the intervention clinic and 304 patients in the control clinic) and 263 had left ICHS or no longer met our age criteria by the conclusion of the intervention. Over the same timeframe, 507 new patients became eligible. In addition, 14 patients from the intervention site switched their care to the control site, while 12 patients from the control site switched to the intervention site.

More than half of the patients in our study sample were 50 to 64 years old and female (Table 2). Over 80% had insurance at baseline, but this proportion decreased to 70% post-intervention. Most patients had a primary care provider with over half having a language-concordant provider.

**Table 2:** Patient demographics

		**Control clinic**		**Intervention clinic**		**Control vs. intervention P value**	
		**Baseline**	**Follow-up**	**Baseline**	**Follow-up**	**Baseline**	**Follow-up**
**Age**	50-64	310 (75%)	386 (75%)	452 (75%)	582 (78%)	0.88	0.23
	65+	102 (25%)	128 (25%)	152 (25%)	164 (22%)		
**Gender**	Female	280 (68%)	353 (69%)	404 (67%)	484 (65%)	0.72	0.16
	Male	132 (32%)	161 (31%)	200 (33%)	262 (35%)		
**Insurance status**	None	61 (15%)	165 (32%)	83 (14%)	213 (29%)	0.15	0.17
	Public	292 (71%)	287 (56%)	406 (67%)	419 (56%)		
	Private	59 (14%)	62 (12%)	115 (19%)	114 (15%)		
**Visits in previous 12 months**	1-2	71 (17%)	156 (30%)	81 (13%)	183 (25%)	0.04	0.07
	3-4	64 (16%)	84 (16%)	73 (12%)	137 (18%)		
	≥ 5	277 (67%)	274 (53%)	450 (75%)	426 (57%)		
**Primary care provider (PCP)**	None	14 (3%)	15 (3%)	4 (1%)	54 (7%)	<0.001	<0.001
	MD	142 (34%)	162 (32%)	440 (73%)	481 (64%)		
	PA/ARNP	256 (62%)	337 (66%)	160 (26%)	211 (28%)		
**Language-concordant PCP**	Yes	215 (52%)	270 (53%)	277 (46%)	325 (44%)	<0.001	<0.001
	No	183 (44%)	229 (45%)	323 (53%)	367 (49%)		
	n/a	14 (3%)	15 (3%)	4 (1%)	54 (7%)		
**Continuity index**	1 visit	39 (9%)	102 (20%)	44 (7%)	91 (12%)	<0.001	<0.001
	Lower	193 (47%)	26 (5%)	465 (77%)	198 (27%)		
	Higher	180 (44%)	386 (75%)	95 (16%)	457 (61%)		
**Charlson-Deyo Comorbidity Score**	0	296 (72%)	363 (71%)	424 (70%)	525 (70%)	0.83	0.99
	1	87 (21%)	113 (22%)	133 (22%)	165 (22%)		
	≥ 2	29 (7%)	38 (7%)	47 (8%)	56 (8%)		

At baseline, Vietnamese patients at the intervention and control clinics had similar overall CRC screening adherence rates (Table 3). During the study period, both sites experienced a decrease in FOBT. The intervention clinic had a 3% increase in overall adherence rate, whereas the control clinic saw no change. Colonoscopy increased during the intervention period, with a greater increase at the intervention clinic compared to the control clinic over time (Table 4); however, the difference of the increase was not statistically significant.

**Table 3:** Colorectal cancer screening adherence at intervention and control clinics at baseline and follow-up

	**Control clinic**		**Intervention clinic**	
	**Baseline (N = 412)**	**Follow-up (N = 514)**	**Baseline (N = 604)**	**Follow-up (N = 746)**
**FOBT**	69 (17%)	47 (9%)	148 (25%)	120 (16%)
**Sigmoidoscopy**	3 (1%)	3 (1%)	16 (3%)	10 (1%)
**Colonoscopy**	98 (24%)	154 (30%)	131 (22%)	250 (34%)
**CRC screening adherence**	158 (38%)	195 (38%)	254 (42%)	338 (45%)

**Table 4:** Pre-post odds ratio estimates using generalized linear mixed models for changes in CRC screening adherence at each clinic*

	**Control clinic**	**Intervention clinic**	**Clinic comparison**	
	**OR (95% CI)**	**OR (95% CI)**	**OR ratio (95% CI)**	**p-value**
**FOBT**	0.54 (0.35, 0.84)	0.77 (0.53, 1.10)	1.42 (0.84, 2.39)	0.19
**Sigmoidoscopy**	1.00 (0.19, 5.38)	0.60 (0.22, 1.61)	0.60 (0.10, 3.72)	0.58
**Colonoscopy**	1.87 (1.32, 2.64)	1.89 (1.27, 2.80)	1.38 (0.89, 2.13)	0.15
**CRC screening adherence**	1.30 (0.95, 1.77)	1.85 (1.44, 2.53)	1.42 (0.95, 2.15)	0.06

*Adjusted for age, gender, insurance status, primary care provider category, language concordance with primary care provider, and continuity index.

Our secondary analysis of patients who were non-adherent to CRC screening at baseline showed a significant increase in overall CRC screening adherence (adjusted OR = 1.70; 95% CI 1.05, 2.75) at the intervention clinic compared to the control clinic (Table 5). The increase in FOBT adherence was marginally-statistically significant (adjusted OR = 1.77; 95% CI 0.98, 3.18).

**Table 5:** Post-intervention CRC screening adherence in cohort who were non-adherent at baseline in each clinic

	**Control clinic (n = 167)**	**Intervention clinic (n = 234)**	**Clinic comparison (N = 401)**	
	Adherence* %	Adherence* %	**OR (95% CI)**	**p-value**
**FOBT**	14.2	22.6	1.77 (0.98, 3.18)	0.06
**Sigmoidoscopy**	na	na	na	na
**Colonoscopy**	20.6	24.9	1.28 (0.74, 2.20)	0.38
**CRC screening adherence**	34.5	47.3	1.70 (1.05, 2.75)	0.03

*Adjusted for age, gender, insurance status, primary care provider category, language concordance with primary care provider, and continuity index. Reported rates are estimated assuming adjustment variable coefficients that are proportional to the observed margins within the non-adherent subsample.

na = Adjusted estimates are not provided due to sparseness of outcome events within data cohort.

Among the 352 patients adherent to CRC screening at baseline and who were also in the post-intervention sample (214 in the intervention clinic and 138 in the control clinic), 79.4% in the intervention clinic and 82.6% in the control clinic were adherent at follow up (OR = 0.81, p = 0.46).

## Discussion

To accelerate the translation of research into practice and mitigate health disparities, EBIs must be effectively adapted for the diverse US population. By simplifying our EBI through a change in content (using a different intervention agent who did not provide health education) and a reduction in intensity (distributing intervention materials only) to fit existing clinical practice and personnel [18], we expanded its reach at a community health center serving a diverse patient population.

Our findings indicate that an EBI, adapted with guidance from conceptual and theoretical frameworks, can increase overall CRC screening among non-adherent patients. This finding is consistent with the general notion that theory-based interventions are more likely to be effective than interventions that do not use a theory or model for planning and evaluation [32]. However, of note Rabin *et al.*’s review of dissemination and implementation studies in community settings found that studies with little or no evidence of effectiveness more often used theory or a conceptual model [33]. Additional research is needed to determine whether theory or conceptually guided adaptations are more effective in clinical than community settings, and whether other differences in these settings exist. This research, which was conducted in a health care setting, must be interpreted accordingly.

A major advantage of our adapted intervention was that no additional staff was needed to implement it. In a recent qualitative study of primary care providers, administrators, and staff, Naughton *et al.* found that adapting the role of MAs enhanced the practice’s ability to achieve patient-centered medical home standards and quality improvement [34]. The authors note that MAs can augment the capacity of physicians and nurses by moving into newly developed practice roles such as health coaches.

We adapted our EBI to match the MA’s roles and responsibilities as well as their workflow [35]. MAs did not need to recruit, obtain patient informed consent, or complete any research documents. To our knowledge, this study is one of the first to evaluate MAs promoting CRC screening during a primary care visit [36]. A randomized study showed that telephone calls from MAs were more efficacious for mammography screening than physician telephone calls or controls [37]. Another study by Ferrer *et al*. used MAs to encourage patients in primary care to quit smoking, quit risky drinking, eat at least five servings of fruits and vegetables per day, and increase physical activity [30]. MAs did not significantly change any risky behaviors in the study by Ferrer *et al.* An ongoing randomized controlled trial of MAs as health coaches for uncontrolled diabetes, hypertension, and hyperlipidemia will provide additional data to guide future interventions and programs [38].

A key strength of our study is the extraction of outcome data from medical records rather than from self-report. FOBT, sigmoidoscopy and colonoscopy follow the same documentation protocols at both clinics and the MAs do not enter any of this information into the EMR. Using a control site within the same EMR system also eliminated potential confounders that might arise when outcome data are extracted from different EMR systems, or when the same EMR system undergoes different upgrades (*i.e.*, both types and timing). In addition, initiatives to promote CRC screening (*e.g.*, Washington State Breast, Cervical and Colorectal Cancer Health Program) were implemented simultaneously at both clinics, eliminating another potential source of confounding.

Our study has several potential limitations. First, this research was conducted in a community health center that serves mostly first-generation Asian immigrants. Accordingly, clinic staff is trained to serve LEP populations, and this may not be the case at other community or private clinics, limiting the generalizability of our results. Second, our quasi-experimental design is unable to confirm a causal relationship between the intervention and screening outcomes. However, we focused on the real-world implementation of an adapted EBI, and experts have recommended similar study designs for dissemination and implementation research [13]. Third, although medical records are considered the gold standard for patient data, the comprehensiveness of medical records from one organization depends on the accuracy with which they document health services received from outside organizations. Our study may therefore underestimate CRC screening in this patient population, in the unlikely scenario where a significant proportion of Vietnamese patients seek primary care and CRC screening from multiple organizations. Fourth, because colonoscopies are referred to specialists outside the community health center, we relied solely on documentation entered into EMR fields by the providers and colonoscopy reports scanned into the EMR system. Fifth, patients at the intervention and control clinics had statistically significant differences in: the type of primary care provider; language concordance with their primary care provider; and continuity index. We adjusted for these variables to maximally balance differences between the two study groups; however, our results must be interpreted within the limitations of the quasi-experimental design. Sixth, unlike primary care providers, patients are not assigned to specific MAs, therefore the EMRs do not have information regarding which MAs were assigned to which patients during their visits. In response to our community health center partner’s needs to provide clinical services in the busy real world, we designed our intervention to minimize disruptions to clinic workflow as well as staff time needed for the research. Therefore, we did not ask clinic staff to document which MA ended up working with which study patient during their visits. Since we did not collect MA-level outcomes, our analysis only accounted for clustering of patients under their primary care providers by GLMMs. Lastly, differentiation of screening from diagnostic colonoscopy data is not available from the current EMR system at ICHS and would require manual audits of 1,260 patient EMR charts. Since manual EMR audits were beyond the scope of our study we erred on the conservative side by including all colonoscopy data and overestimated the rate of screening colonoscopy.

By conducting this research in the real world, we experienced changes in the clinic setting that further diminished the intervention’s intensity. When the proposal for this study was submitted, MAs at ICHS had greater cultural and linguistic congruence with their patients, an intended aspect of compatibility. By the time our study was implemented, however, staffing changes with certified MAs who were also proficient with EMRs reduced cultural and linguistic congruence with the LEP patients. Additionally, significant staff turnover in the intervention clinic (almost 90% of total clinic staff, including MAs) occurred during the study. Given its reduced intensity (both intended and unintended), the effect of the adapted intervention was significantly less than that of the original EBI with a health educator [22]. We surmise that the adapted intervention effect would have been greater if the MAs had better cultural and linguistic congruence with their patients since both can be barriers to conveying the importance of CRC screening. Similarly in settings with less staff turnover the adapted intervention may have had a greater effect; however, staff turnover could potentially influence the results in unpredictable ways.

On the other hand, data compiled by ICHS suggest that the effect of our adapted intervention may have extended beyond the Vietnamese patients targeted in this study. Based on 2011 data from the Healthcare Effectiveness Data and Information Set, 62% of age-eligible patients at the intervention clinic with commercial insurance, and 64% with Medicare, had appropriate CRC screening, compared to 48% and 44%, respectively, at the control clinic. This pattern was not duplicated with other types of cancer screening at the two clinics.

## Conclusions

Although this research only demonstrated our adapted intervention to be efficacious in overall CRC screening adherence, the study provides a strategy to guide the adaptation of EBIs for broader reach, in particular hard to reach LEP populations. As recommended by Diffusion of Innovations theory, in modifying our EBI, we focused on the influential attributes and the communication channel. The relative ease with which our adapted intervention was incorporated into clinic activities holds promise for broader implementation in busy primary care settings and may promote the feasibility and sustainability of EBIs. Effective adaptation of EBIs must be further studied in order to mitigate the health disparities of hard-to-reach populations in a timely manner.

## Competing interests

The authors declare that they have no competing interests.

## Authors’ contributions

SPT conceived of the study, carried out the study, and prepared the manuscript. AC participated in the inception and implementation, of the study as well as the interpretation and study results. YY was the senior biostatistician and oversaw the study design and analysis. AK conducted the data analysis and contributed to the manuscript. MPY participated in the implementation of the study. VT participated in the design and interpretation of the study and its results. RB participated in the design and interpretation of the study and its results. All authors read and approved the final manuscript.
